# Brugada Syndrome: The Role of Risk Stratification in Selecting Patients for Implantable Cardioverter-defibrillator Placement

**DOI:** 10.7759/cureus.2799

**Published:** 2018-06-13

**Authors:** Michael Mankbadi, Samira Hassan, Michelle McGee, Bonnie Jan, Sibani Mangal, Jake Altier, Manjunath Harlapur

**Affiliations:** 1 Other, University of Central Florida College of Medicine, Orlando, USA; 2 Cardiology, Bay Pines VA Healthcare System, Bay Pines, USA

**Keywords:** brugada syndrome, brugada, electrophysiology, sudden cardiac death, channelopathy, genetics, syncope, cardiac arrhythmias, genetic diseases, implantable cardioverter-defibrillator device

## Abstract

Brugada syndrome (BS) is an inherited cardiac ion channelopathy that is a rare, but treatable, cause of sudden cardiac death (SCD). There are many studies that explore the management of symptomatic BS, but few trials have been conducted regarding management of asymptomatic Brugada patients. Asymptomatic BS patients are shown to be at increased risk (0.5%-1.5%) for SCD compared to the general population and account for nearly 20% of deaths from SCD in patients with structurally normal hearts. Treatment for asymptomatic BS patients is often debated with the current guidelines allowing for management decisions to be made on a case-by-case basis. Therapies include either anti-arrhythmic medications, implantable cardioverter-defibrillator (ICD) placement, or no active treatment. This review intended to assess whether ICD placement benefits asymptomatic BS patients and what criteria may be useful in selecting patients for ICD placement. Results showed that ICD placement can reduce mortality in select asymptomatic patients. There were certain risk factors that increased the likelihood that an asymptomatic patient would experience SCD and thus benefit from an ICD. These factors include an electrocardiogram(ECG) demonstrating spontaneous type 1 Brugada Syndrome and inducibility of ventricular tachyarrhythmias during electrophysiological study. Other variables including gender, family history of SCD, and the presence of SCN5A mutation were not predictive of arrhythmic events. Moreover, many patients can suffer complications from ICDs that can affect the quality of life including inappropriate shocks, device malfunction, infection, mental health problems, and difficulties with replacements. Guidelines for quantifying the risk of SCD relative to the risks associated with ICD placement are still poorly defined. These complications and risk factors should be taken into consideration in the context of a patient-centered discussion regarding ICD placement in asymptomatic patients.

## Introduction and background

Brugada syndrome (BS) is a genetically inherited arrhythmogenic disease characterized by abnormal electrocardiogram (ECG) findings and may cause sudden cardiac death (SCD). Three types of ECG findings have been described in Brugada syndrome. Type 1 is the classical type with typical ECG findings of a J elevation of 2 mm (0.2 mV), a coved-type ST-segment followed by a negative T wave in one of right precordial leads (V1-V3). Other diagnostic criteria include documented ventricular fibrillation or polymorphic ventricular tachycardia, family history of SCD < 45 years old, coved-type ECGs in family members, inducible VT, syncope, and/or nocturnal agonal respiration. Types 2 and 3 are non-diagnostic but generally warrant investigation. Type 2 is characterized by a gradually descending ST-segment elevation (remaining ≥1 mm above the baseline), followed by a positive or biphasic T-wave that results in a saddle-back configuration. Type 3 can be the morphology of either type 1 or 2 but with <2 mm of ST elevation in the right precordial leads [1,2]. However, as BS can clinically manifest as a sudden cardiac arrest or have intermittent ECG pattern, it is clinically difficult to diagnose. Currently, utilization of a pharmacological challenge with antiarrhythmic class I drugs is used in an attempt to reveal abnormal ECGs.

BS is believed to be due to genetic abnormalities resulting in a channelopathy, although in the majority of patients, no mutation has yet been identified. The most commonly implicated gene is the SCN5A gene which is found in 20% of patients and encodes for a sodium channel [3]. Due to higher penetrance, it is more often seen in males. The prevalence of  Brugada type 1 ECG appears more frequently in Asia (0%–0.36% of the population) and Europe (0%–0.25%) than in the United States (0.03%). On the other hand, type 2 and type 3 ECGs are more prevalent in Asia (0.12%–2.23%) than in Europe (0.0%–0.6%) or the United States (0.02%) [4]. Regardless of its low prevalence, it is considered a major cause of sudden unexplained death syndrome (SUDS). It can also significantly shorten a patient’s life, with the average age of death being 41 [5].

Clinically, BS can present with syncope, palpitations, SCD, or asymptomatic screening. The cause of death in patients with BS is linked to ventricular fibrillation. These arrhythmias can often occur without warning including commonly during sleep. There is currently no exact treatment to totally prevent ventricular fibrillation (VF). Recent studies have identified quinidine, an antiarrhythmic drug, as a potential treatment that decreases the likelihood of VF [6]. Moreover, it is important for patients with BS to avoid certain medications which may induce an arrhythmia. In order to potentially decrease the risk of SCD, one treatment modality involves insertion of an implantable cardioverter-defibrillator (ICD) which can cardiovert an episode of VF. However, there has been insufficient evidence to accurately assess whether this treatment modality in asymptomatic patients improves patient outcomes. Currently, there is no agreed-upon criteria to stratify asymptomatic patients into treatment groups. Thus, this paper investigates the current literature to determine whether ICD implantation benefits patients with asymptomatic BS and whether any criteria can be used to stratify patients based on risk.

## Review

We reviewed 17 articles that were predominantly case-controlled and retrospective studies. There have been no randomized control studies on ICD placement in asymptomatic patients with BS. The prevalence of BS is low which makes randomized control studies difficult to undertake and decreases the statistical power of the study results. There is much controversy as to the risk stratification of patients with asymptomatic BS, and the following studies compare and contrast different approaches to determine the necessity of ICD placement in these patients.

FINGER study

In 2010, 1029 patients from the FINGER (France, Italy, Netherlands, Germany) Brugada syndrome registry were studied to determine and evaluate the prognosis and risk factors of SCD in BS patients [7]. This sample included 654 asymptomatic patients, 313 with prior syncope, and 62 with prior events of SCA with a median age of 45 years. These patients were followed for a median of 32 months. The clinical data of interest for this study included age, gender, circumstances at diagnosis, and family history of SCD. Based on ECG abnormalities, three major groups of subjects were identified: subjects after an episode of aborted SCD, subjects during diagnostic evaluation of syncope, and asymptomatic subjects who had a type 1 or a suspicious ECG during routine examination or family screening. Electrophysiological study (EPS) was performed on 638 (62%) subjects, and the rate of inducible ventricular tachyarrhythmia was higher in previously symptomatic patients (46%) than in asymptomatic patients (37%). Moreover, as expected ICD implantation rate was higher in symptomatic patients at 69.9% than in asymptomatic patients at 26.1%. During the follow-up, 35% of patients from the SCD group, 6% of patients from the syncope group, and 1.5% of patients from the asymptomatic group had experienced an arrhythmic event.

Analysis of asymptomatic patients showed that neither spontaneous type 1 ECG, male gender nor age was predictive of a shorter time to the first arrhythmic event during follow-up, while patients with positive EPS had a shorter time to first arrhythmic event. If introduced in the multivariable analysis of asymptomatic patients, ICD implantation was found to be a predictor of a shorter time to first arrhythmic event. Overall, the occurrence of arrhythmic events in asymptomatic patients is low with an event rate of 0.5% per year and SCD occurring in two of 478 asymptomatic patients who did not receive ICD implantation. Due to this low rate, there exists difficulty in identifying a standardized therapeutic approach for asymptomatic patients. Currently, ICD implantation is the only effective means of preventing SCD in BS patients, but it has been shown recently that the incidence of ICD-related complications is high and deaths related to ICD malfunction have also been reported [7]. It was concluded that patients with either previous SCA or a history of syncope were much more likely to experience future arrhythmic events, while asymptomatic patients had a very low risk of experiencing future arrhythmic events. The presence of symptoms and spontaneous type 1 ECG were considered predictors of arrhythmic events. However, other factors such as gender, familial history of SCD, inducibility of ventricular tachyarrhythmias during electrophysiological study, and the presence of an SCN5A mutation were not predictive of arrhythmic events.

Risk stratification

It is well accepted that symptomatic BS patients are at high risk of recurrent VF episodes and thus have the strong (gold standard) indication for ICD placement. However, no criteria exist for identification of the high-risk patients within the asymptomatic BS subset. While it is agreed that the risk of SCD in asymptomatic patients remains low, 0.5%-1.5% per year, the concern surrounds the high risk of death secondary to the development of VF in a patient who does not have an ICD implant [1, 2]. With this in mind, there are few general management recommendations offered for asymptomatic patients. There is growing research into the utility of risk stratification to help better identify possible risk factors to predict SCD in this specific population of patients [1, 2, 8]. Much of the current literature has agreed that aborted SCD, syncope, and spontaneous ECG pattern are conventional risk variables that confer high risk of SCD in BS patients [7, 9-11]. Given the genetic correlation in BS, it was thought that stratifying patients based on family history of SCD, SCN5A mutations, inducibility of ventricular tachyarrhythmias during EPS, and gender could further help to identify high-risk patients, however, multiple studies have not found a significant association [7, 11].

One study at Okayama University Hospital evaluated symptomatic and asymptomatic patients to determine potential ECG markers that can help with risk stratification of asymptomatic patients [12]. The study consisted of 471 patients diagnosed with Brugada syndrome based on the criteria of Expert Consensus Statements. Patients were divided according to symptoms at the initial visit to the hospital, 326 patients who did not experience syncope and 145 who experienced syncope or had a VF episode. The following ECG markers were analyzed: RR, PQ, QRS, QT, and Tpe intervals, ST level, atrial fibrillation, AV block, spontaneous type 1 ECG, early repolarization, and fragmented QRS. Univariable analysis showed that QRS width, Tpe interval, ST level and fQRS were common risk factors for the initial and recurrent ventricular tachyarrhythmic events and that intervals of QT and QTa, ER and AF were predictors for VT recurrence. Multivariable analysis of the markers in the symptomatic group showed that fQRS, Tpe, and ER were independent risk factors for recurrent episodes. They did not evaluate multivariable analysis in the asymptomatic group because of low event ratio in this group. Intervals of fQRS and Tpe interval are risk factors for initial events in both groups, whereas ER was a predictor for recurrent events. These results indicate that risk factors for the initial ventricular tachyarrhythmic event and those for recurrent ventricular tachyarrhythmic events are different. This is a retrospective study and thus subjected to selection bias, especially in patients who were consulted to the Okayama University Hospital. The study findings should be taken as preliminary findings that need further investigation such as a prospective study with a larger sample size.

In another study, authors evaluated ICD placement in BS patients over the span of 20 years [11]. They evaluated a number of risk factors, including family history, sex and arrhythmia inducibility in the determination of ICD use in asymptomatic patients. Of 176 patients, 118 were male (67%), 46 were completely asymptomatic (26.1%) and 27 had a family history of Brugada Syndrome (15.3%). Ventricular arrhythmia inducibility was found to have a hazard ratio of 2.44, with a confidence interval (CI) of 1.18 to 6.02 (p = 0.04). Together, these factors, family history, sex, and arrhythmia inducibility were noted to show an increase in the occurrence of appropriate shocks in the follow-up period. The authors concluded that these three elements should be considered when doing a risk assessment [11]. Though this study was able to stratify ICD implantation over a long time period, the variability and evolution in technique for assessment, management and follow-up put forth an inevitable limitation. Further evaluations of these risk factors should be assessed in a larger population over a longer time period.

When stratifying risks, it is generally accepted that all symptomatic patients should receive ICD implantation. However, it has been noted that syncope, one of the hallmark symptoms of BS, may play a controversial role in assessing whether or not a patient should be given an ICD. In one evaluation of ICD placement in BS patients, the author noted that it can be difficult to confirm the true origin of syncope in every case of BS. A study assessing 88 BS patients who reported syncope as their primary symptom showed that nine patients (10%) experienced appropriate shocks, but 7% had a recurrence of their syncope without any record of arrhythmia after ICD implantation [2]. This discrepancy further demonstrates how difficult it can be to identify high-risk asymptomatic patients, especially those who may have had an episode of syncope in the past.

Additionally, various studies have implied that asymptomatic patients have a low risk of developing arrhythmic events, which is why risk stratification as a means of determining treatment and intervention in asymptomatic BS patients is still controversial [13-15]. One study recommended against ICD placement in asymptomatic patients with inducible type I Brugada ECG and no family history of SCD as the risk of SCD was significant low [16].

Even with the identification of significant risk factors, a population-based risk stratification system will be challenging to implement at an individual level. We recommend that ICD placement in asymptomatic patients be made on a case-by-case basis in the context of a patient-centered discussion. All patients, no matter their individual risk assessment, should implement specific lifestyle changes to further lessen their estimated risk of VT associated SCD, including: avoiding proarrhythmic drugs, alcohol, and cooling fevers quickly with antipyretic agents [11, 13]. Furthermore, it is imperative that physicians discuss with their patients the limitations of our current knowledge and the risks/benefits of treatment.

Complications of ICD implantation

ICD implantation poses a number of complications for patients both operatively and post-operatively. The most widely noted complication of ICD placement across multiple studies is the risk of inappropriate shocks. Inappropriate shocks were defined in one study as those that were delivered without the presence of any ventricular arrhythmias, usually in response to lead failure or dislodgement, T wave oversensing, device failure, sinus tachycardia, or supraventricular tachycardia [11]. One retrospective analysis of 176 patients diagnosed with Brugada syndrome and treated with ICD implantation found that 18.7% of the patients being reviewed experienced inappropriate shocks, with a mean of 3.0 +/- 5.7 shocks per patient [17]. In another study, Rosso et al. assessed 59 patients with Brugada Syndrome and subsequent ICD implantation; 13.5% of these patients required psychiatric attention after their ICD implants caused certain complications, including inappropriate shocks (27.1%), device malfunction (16.9%) and infection (1.7%). The implantation was shown to impact the quality of life and ultimately mental health, especially in younger patients who were more likely to undergo device replacements and extractions later in life [11].

Other concerning complications are those that can happen due to the implantation itself. In the Rosso et al. study, complications included pneumothorax, brachial plexus injury, and right ventricular perforation with subsequent pericardial effusion in three separate patients [11, 18-23 ]. In his study, Conte described events of cardiac arrest, myocardial infarction, septic shock, and lethal arrhythmias shortly after device implantation. The lethal arrhythmia was an episode of ventricular fibrillation that was unresolved by both internal and external defibrillations. It is important to note, however, that these patients were symptomatic prior to device implantation. Those who were labeled as asymptomatic faced complications such as syncope (6.5%), and paroxysmal atrial fibrillation (13.0%) in the long-term follow-up period [17,18]. When stratifying the benefits and risks of implanting an ICD in asymptomatic patients, it is important to keep in mind the profound medical and psychological impacts that the procedure can have on them.

## Conclusions

Many studies demonstrate that ICD placement in asymptomatic patients does reduce mortality, with one study showing 0% mortality at 10-year follow-up. However, ICD placements were also shown to have many complications including inappropriate shocks, device malfunction, infection, and psychological burden. The risk of an asymptomatic patient experiencing an arrhythmic event is rather low at 0.5%-1.5% over an average of 73 months. Risk factors for SCD in the asymptomatic patient include an ECG demonstrating spontaneous type 1 Brugada Syndrome and inducibility of ventricular tachyarrhythmias during electrophysiological study. Other risk factors including gender, family history of SCD, and the presence of SCN5A mutation were not predictive of arrhythmic events. ECG findings such as fQRS and Tpe have also been associated with increased risk of initial VTA events. Further investigation is needed to stratify asymptomatic patients based on the risk of arrhythmic events in order to determine which patients would benefit from ICD placement.
